# Migraine remains second among the world’s causes of disability, and first among young women: findings from GBD2019

**DOI:** 10.1186/s10194-020-01208-0

**Published:** 2020-12-02

**Authors:** T. J. Steiner, L. J. Stovner, R. Jensen, D. Uluduz, Z. Katsarava, on behalf of Lifting The Burden: the Global Campaign against Headache

**Affiliations:** 1 Department of Neuromedicine and Movement Science, NTNU Norwegian University of Science and Technology, Edvard Griegs gate, Trondheim, Norway; 2 Division of Brain Sciences, Imperial College London, London, UK; 3 Norwegian Advisory Unit on Headache, Department of Neurology and Clinical Neurophysiology, St Olavs University Hospital, Trondheim, Norway; 4 Danish Headache Centre, Department of Neurology, University of Copenhagen, Rigshospitalet Glostrup, Glostrup, Denmark; 5 Neurology Department, Cerrahpaşa School of Medicine, Istanbul University, Istanbul, Turkey; 6 Evangelical Hospital Unna, Unna, Germany; 7 Department of Neurology, University of Duisburg-Essen, Essen, Germany; 8 EVEX Medical Corporation, Tbilisi, Georgia; 9 IM Sechenov First Moscow State Medical University (Sechenov University), Moscow, Russian Federation

The capstone papers on the Global Burden of Disease study 2019 (GBD2019), delayed by a diversion of resources to mapping covid-19, appeared in *Lancet* on October 17th. The accompanying announcement by the Institute for Health Metrics and Evaluation (IHME) described GBD2019 as “the largest and most comprehensive effort to quantify health loss across places and over time”, including “more than 3.5 billion estimates of … 369 diseases and injuries … in 204 countries and territories” [1].

IHME had previously announced its move to a 3-year cycle of major model updates for most non-fatal causes and risk factors (but not causes of death) [2]. Each future GBD round will include a subset of these “in rotation”, while still producing results each year for all causes of death and all non-fatal outcomes. The focus among the capstone papers was therefore on disability-adjusted life years (DALYs) [3], without the usual separately reported estimates of years lived with disability (YLDs). From a policy perspective (GBD’s main purpose is to inform health policy), this makes complete sense: years of healthy life lost to early mortality are clearly no less important than those lost to disability. But the approach takes away the spotlight from disabling diseases that do not cause early death – such as headache disorders.

Nevertheless, headache disorders in 2019 ranked 14th among global causes of DALYs (all ages, both genders) [3]. Seven non-communicable disorders were ranked higher: ischaemic heart disease, stroke, chronic obstructive pulmonary disease, diabetes, low back pain, congenital defects and depressive disorders [3]. Among females, headache disorders were tenth, below gynaecological diseases (ninth) but above depressive disorders (11th). Among young adult females (15–49 years), they were second only to gynaecological diseases (note that this was of DALYs, not YLDs). Among young adult men they were tenth, with road injuries, self-harm, interpersonal violence and cirrhosis – all causes of premature mortality – each responsible for more DALYs.

What about YLDs? In separate on-line estimates, headache disorders were the cause in 2019 of 46.6 million YLDs globally, 5.4% of total YLDs, with 88.2% of these attributable to migraine [4]. In terms of lost healthy life, that equates to 46.6 million people dying one year early. In the ranked causes of YLDs (all ages, both genders), headache disorders (602.5 per 100,000 person/years) were third, below low back pain (823.0) and, by a tiny margin, depressive orders (605.7) (Table 1). Among females, gynaecological diseases (second: 764.0) overtook both headache (third: 751.0) and depressive disorders (fourth: 743.7) (Table 1) despite their clearly evident association with female gender. Also clearly evident was the association of headache disorders with age – specifically, with young adulthood. Among females aged 15–49 years, headache disorders (1016.1) were second only to gynaecological diseases (1230.5), with depressive disorders third (890.4). But in all young adults, with gynaecological diseases a factor among only half, headache disorders (813.4) were top cause of YLDs (Table 1).

**Table 1:** GBD2019: Top level-3 causes of global disability (expressed as years lived with disability [YLDs]) by gender and age (data from [3, 4])

Gender	Age range (years)	Rank	Cause	% of total YLDs [uncertainty interval]
Both	All	1	Low back pain	7.4 [6.2–8.7]
		2	Depressive disorders	5.5 [4.3–6.8]
		3	**Headache disorders**	5.4 [1.1–10.6]
	15–49	1	**Headache disorders**	8.0 [1.6–15.7]
		2	Low back pain	7.6 [6.1–9.3]
		3	Depressive disorders	7.3 [5.7–9.2]
Male	All	1	Low back pain	7.0 [5.8–8.2
		2	Age-related hearing loss	5.2 [4.3–6.4]
		3	Diabetes	4.9 [4.2–5.7]
		4	Depressive disorders	4.7 [3.7–5.9]
		5	**Headache disorders**	4.6 [1.0–9.0]
	15–49	1	Low back pain	7.8 [6.3–9.6]
		2	**Headache disorders**	7.0 [1.5–13.8]
		3	Depressive disorders	6.6 [5.2–8.3]
Female	All	1	Low back pain	7.7 [6.5–9.2]
		2	Gynaecological diseases	6.2 [5.1–7.3]
		3	**Headache disorders**	6.0 [1.2–12.0]
		4	Depressive disorders	6.0 [4.8–7.5]
	15–49	1	Gynaecological diseases	10.7 [8.7–12.9]
		2	**Headache disorders**	8.7 [1.6–16.2]
		3	Depressive disorder	7.8 [6.0–9.9]

There were variations according to World Bank region and country income level. Headache disorders were third cause of YLDs in East Asia & Pacific and in Middle East & North Africa, but second in Europe & Central Asia, fourth in South Asia and in sub-Saharan Africa, fifth in Latin America & Caribbean and (surprisingly) sixth in North America. They were third in countries classed by the World Bank as lower- or upper-middle-income, but fourth in low-income countries and fifth in those classed as high income. The association between headache and socioeconomic status has never been clear!

GBD is wholly dependent on data. It applies highly sophisticated modelling to fill data gaps, “borrowing strength between locations and over time” [3]. But extrapolations from nearby countries to those where data are sparse or totally lacking is a process that cannot be free from uncertainty (evidenced by the wide uncertainty intervals around estimates for headache disorders [Table 1]). Not too much should be made of these variations.

The level-3 grouping of headache disorders in GBD2019 includes only specific diseases: migraine and tension-type headache (TTH), each with medication-overuse headache (MOH) as a sequela factored in according to the proportion of MOH attributed to it [3]. Low back pain, on the contrary, is a symptom. It ought to, and hopefully will in future iterations of GBD, be split according to its diverse aetiologies. Even at level 4 in IHME’s analyses – supposedly of specific disorders – low back pain remains as a listed single cause of YLDs, and inevitably is ranked first among all but young adult women (Table 2). Migraine remains second overall (both genders, all ages) but takes first place in young women as it did in GBD2016 [5] (Table 2). In fact, migraine is top cause of DALYs in young women (Table 3), a finding, surely, of profound significance. No other disease, communicable or non-communicable, is responsible for more years of lost healthy life in young women, notwithstanding that migraine causes no premature mortality.

**Table 2:** GBD2019: Top level-4 causes of global disability (expressed as years lived with disability [YLDs]) by gender and age (data from [3, 4])

Gender	Age range (years)	Rank	Cause	% of total YLDs [uncertainty interval]
Both	All	1	Low back pain	7.4 [6.2–8.7]
		2	**Migraine**	4.9 [0.8–10.1]
		3	Age-related hearing loss	4.7 [3.8–5.7]
	15–49	1	Low back pain	7.6 [6.1–9.3]
		2	**Migraine**	7.3 [1.1–15.1
		3	Major depression	5.8 [4.3–7.5]
Male	All	1	Low back pain	7.0 [5.8–8.2
		2	Age-related hearing loss	5.2 [4.3–6.4]
		3	Diabetes type 2	4.7 [4.0–5.4]
		4	**Migraine**	4.1 [0.7–8.3]
	15–49	1	Low back pain	7.8 [6.3–9.6]
		2	**Migraine**	6.3 [1.1–12.8]
		3	Major depression	5.2 [3.8–6.7]
Female	All	1	Low back pain	7.7 [6.5–9.2]
		2	**Migraine**	5.5 [0.9–11.6]
		3	Other musculoskeletal	5.0 [3.8–6.4]
	15–49	1	**Migraine**	8.0 [1.2–16.7]
		2	Low back pain	7.4 [5.9–9.1]
		3	Major depression	6.2 [4.6–8.2]

**Table 3:** GBD2019: Top level-4 causes of global lost healthy life (expressed as disability-adjusted life years [DALYs]) among young adult women (data from [3, 4])

Rank	Cause	% of total DALYs [uncertainty interval]
1	**Migraine**	4.9 [0.7–10.6]
2	Low back pain	4.5 [3.4–5.6]
3	Major depression	3.8 [2.7–4.9]

New to GBD2019 were bias adjustments to make allowance for low-quality sampling and survey methods, and for a range of other methodological deficiencies in data sources [4]. This is an important development, since epidemiological methods in headache have improved over the last decade [6], and case definitions have changed over the last two [7, 8]. These, too, were factors contributing to the wide uncertainty intervals. Also as a methodological innovation, GBD2019 took separate account of definite and probable migraine and of definite and probable TTH [8], using individual participant data from studies in 19 countries on frequency and duration of episodes to estimate proportions of time in ictal state for each [4].

The authors of the GBD2019 report wrote: “The prominent position of headache disorders in the DALY rankings in the 10-24-year and 25-49-year age groups has received little attention in global health policy debates” [3]. A similar message has been our repeated *cri de coeur* [5, 9–12]. They added: “While there is no cure for these disorders, there are effective symptomatic and preventive treatments available.” This, of course, is not a revelation in headache circles, but outside them it appears still to be so. Remediability is the crucial issue in claims for priority in health care, especially when there is strong evidence of cost-effectiveness [13]. The disability burden of headache disorders – particularly of migraine, by far the principal contributor [3] – is concentrated among those of productive age. It is this factor that keeps headache high among the causes of YLDs (and DALYs) in less wealthy countries, where shorter life expectancies raise the population proportions of young adults. It is this, also, that adds – or should add – a dimension of mind-focusing concern for policy makers everywhere [5, 14].
